# Applications of crowdsourcing in health: an overview

**DOI:** 10.7189/jogh.08.010502

**Published:** 2018-06

**Authors:** Kerri Wazny

**Affiliations:** Centre for Global Health Research, Usher Institute of Informatics and Population Sciences, University of Edinburgh, Edinburgh, UK

## Abstract

**Background:**

Crowdsourcing is a nascent phenomenon that has grown exponentially since it was coined in 2006. It involves a large group of people solving a problem or completing a task for an individual or, more commonly, for an organisation. While the field of crowdsourcing has developed more quickly in information technology, it has great promise in health applications. This review examines uses of crowdsourcing in global health and health, broadly.

**Methods:**

Semantic searches were run in Google Scholar for “crowdsourcing,” “crowdsourcing and health,” and similar terms. 996 articles were retrieved and all abstracts were scanned. 285 articles related to health. This review provides a narrative overview of the articles identified.

**Results:**

Eight areas where crowdsourcing has been used in health were identified: diagnosis; surveillance; nutrition; public health and environment; education; genetics; psychology; and, general medicine/other. Many studies reported crowdsourcing being used in a diagnostic or surveillance capacity. Crowdsourcing has been widely used across medical disciplines; however, it is important for future work using crowdsourcing to consider the appropriateness of the crowd being used to ensure the crowd is capable and has the adequate knowledge for the task at hand. Gamification of tasks seems to improve accuracy; other innovative methods of analysis including introducing thresholds and measures of trustworthiness should be considered.

**Conclusion:**

Crowdsourcing is a new field that has been widely used and is innovative and adaptable. With the exception of surveillance applications that are used in emergency and disaster situations, most uses of crowdsourcing have only been used as pilots. These exceptions demonstrate that it is possible to take crowdsourcing applications to scale. Crowdsourcing has the potential to provide more accessible health care to more communities and individuals rapidly and to lower costs of care.

The term crowdsourcing was coined a decade ago in Howe’s 2006 *Wired* article and the field of crowdsourcing in academia and industry has since grown exponentially [1-3]. Despite recent interest in the field, the actual practice of crowdsourcing has been in use for hundreds of years [3-5]. Some of the early uses of crowdsourcing include Britain’s Longitude Act and Galton's report of an experiment where 787 people collectively and accurately guessed the weight of an ox [3,6,7].

Crowdsourcing refers to a large group of people collectively solving a problem or completing a task for an individual or an organisation. Its definition is disputed [2,8,9]. The terms citizen science, mHealth, and wisdom of the crowds are often incorrectly used interchangeably with crowdsourcing [9-11]. Crowdsourcing differs from citizen science in that it does not necessarily involve laypersons contributing and it can be using mHealth technology but this is not a requirement. ‘Wisdom of the crowds’ refers to a specific type of crowdsourcing that capitalises on people’s collective knowledge; there are examples of crowdsourcing that do not require people to use knowledge in order to complete tasks. An in-depth exploration of defining crowdsourcing is reviewed elsewhere [8].

The field of crowdsourcing has developed in information technology or business, but crowdsourcing can be a promising tool in health, and in global health in particular. It is rapid, low cost, and can collect a huge amount of information from a large number of people [3,12-15]. It also is a flexible method that has the potential to cover a variety of research, including quickly evolving epidemiological research and traditional behavioural research. It can cover unpredictable events, produce novel discoveries, and can also be used to raise public awareness[3,12-20]. Research in crowdsourcing has also been shown to be at least as accurate as traditional research methods [10,21].

This review examines uses of crowdsourcing in health, and in global health in particular.

## METHODS

This review is the second part of a larger review on crowdsourcing [8]. Previously conducted reviews missed many peer-reviewed papers, due to poor indexing of crowdsourcing [3,22,23]; thus, Google Scholar was used to search peer-reviewed and grey literature for articles related to crowdsourcing and health. Semantic searches were conducted by combining ‘crowdsourcing’ with health-related terms, including ‘health,’ ‘public health,’ ‘genetics,’ and ‘disease,’ etc. A full list of the sematic searches can be found in **Box 1**. Searches were conducted in August 2015 and titles of results were scanned directly from within Google Scholar. Pages of search results within Google Scholar were scanned until it was clear that the results retrieved were no longer relevant. **Box 1** provides details on how many pages of Google Scholar results were searched for each semantic search. 996 articles were identified through the Google Scholar search, of which 375 were discarded as duplicates or as irrelevant (not crowdsourcing) once abstracts were read. 285 of the articles related to various aspects of health. All 285 articles were read and organised into categories; as this is a narrative review, this article reports on the clusters that formed most prominently after organising the articles into categories in order to give an overview of the ways in which crowdsourcing is being used in health.

Box 1 **Crowdsourcing semantic searches conducted in Google Scholar.**

1. “Crowdsourcing”
  - Up to 25 pages
2. “Crowdsourcing” and “Health”
  - Up to 15 pages
3. “Crowdsourcing” and “Immunology”
  - Up to 5 pages
4. “Crowdsourcing” and “Genetics”
  - Up to 9 pages
5. “Crowdsourcing” and “Public Health”
  - Up to 20 pages
6. “Crowdsourcing” and “Disease”
  - Up to 25 pages
7. “Crowdsourcing” and “Surveillance”
  - To 20 pages
8. “Crowdsourcing” and “Diagnosis”
  - Up to page 14

This review provides an overview of some of the ways crowdsourcing has been used, using a selection of the papers identified as illustration.

## RESULTS

Eight areas of importance were identified: (i) diagnosis; (ii) surveillance; (iii) nutrition; (iv) public health and environment; (v) education; (vi) genetics; (vii) psychology; and, (viii) general medicine/other. **Table 1** provides an in-depth description of the individual studies.

**Table 1:** Description of studies included in overview

Reference	Category	Topic	How crowdsourcing is used	Results
**Mavandadi et al. [****24****], Ozcan [****25****]**	Diagnosis	Malaria diagnosis	Uses gaming (BioGames) to diagnose malaria parasites. Gamers are given a tutorial, and must achieve accuracy of >99% in training game before playing real game. Gamers asked to label cell as infected vs healthy.	Gamer diagnoses had an accuracy of 99%, sensitivity of 95.1% and specificity of 99.4%. Authors suggests that gaming could be a viable option for telepathology.
**Feng et al. [****26****]**	Diagnosis	Malaria – education through diagnosis	Based off BioGames app [24,25], used gamification to train non-experts to diagnose malaria parasites and compared diagnoses to experts.	BioGames has achieved diagnostic accuracy comparable to that of experts when scores from individual non-experts are aggregated and the crowd size is large.
**Luengo-Oroz et al. [****27****]**	Diagnosis	Malaria diagnosis	A crowdsourcing game (MalariaSpot) was designed using malaria-positive blood films. Players were asked to tag as many malarial parasites as possible in 1 min and given continuous feedback.	Combination of games or more resulted in extremely accurate identification of malarial parasites (99% accuracy).
**Mitry et al. [****28****]**	Diagnosis	Detecting glaucomatous optic neuropathy	Uses Amazon Mechanical Turk to study viability of crowdsourcing to detect glaucomatous optic neuropathy. Turkers were asked to classify images as normal or abnormal, with each image being classified 20 times.	Authors had two groups, one did not restrict and the other restricted to high-performing Turkers. Sensitivity was high across both, ranging from 83%-88%, but specificity was poor, ranging between 35%-43%.
**Brady et al. [****29****]**	Diagnosis	Grading of diabetic retinopathy	Uses Amazon Mechanical Turk for classifying fundus photos of diabetic retinopathy.	81.3% of images were correctly classified in an average time of 25 s per image. However, Turkers struggled to specify the level of severity.
**do Reis et al. [****30****]**	Diagnosis	Large scale molecular pathology studies in cancer	Used 98 293 citizen scientists to access cell slider web page and score tumor markers. Specifically, citizen scientists scored sub-images of tissue microarray cores labelled for estrogen receptor prognosis.	The citizen scientists performed well in identifying cancer (area under ROC curve 0.95, 95% CI 0.94 to 0.96), and estrogen receptor status (0.97, 95% CI 0.96 to 0.97), and was similar to trained pathologists.
**Gehl et al. [****31****]**	Diagnosis	Skin self-examination for melanoma	Conducted a physical crowdsourcing exercises in a mall, recruiting 500 participants and teaching basic skin self-examination techniques. Implemented various thresholds to improve crowd results.	Using a 19% threshold, 90% of melanomas were identified and 72% of non-melanomas, and with a 65% threshold, 67% of melanomas were identified and 100% of non-melanomas. Authors recommend the 19% threshold.
**Xiang et al. [****32****]**	Diagnosis	Diagnosing medical images	Because there is a lack of high-level experts in rural China, the authors investigated whether crowdsourcing could be used to diagnose medical images. 2^nd^- or 3^rd^-year graduate students with a medical imaging major participated.	The average accuracy was 39.54%, with the best student only making the correct diagnosis 50% of the time. Using a machine learning algorithm with majority voting, combined with crowdsourcing, which learns the students’ mistakes, accuracy can increase to 80%.
**Cheng et al. [****33****]**	Diagnosis	Diagnosing medical illnesses	Investigated the feasibility of using crowdsourcing for diagnosing medical conditions with case descriptions of varying difficulty, posted on Amazon Mechanical Turk, O’Desk, and web forums.	Web forums were ineffective. Turkers diagnosed easy to diagnose cases. O’Desk workers were able to diagnose easy cases, but were more likely to express caution when providing diagnoses for any complicated cases, and some refused to provide diagnoses.
**Sims et al. [****34****]**	Diagnosis	Point-of-care problem solving for clinicians	Authors reports clinicians’ experiences using a crowdsourcing application for point-of-care problem solving, where clinicians post problems via an app and these are answered by verified users and viewable by users in that user’s provider group	Over 80% of respondents felt that app could have a positive impact on patient care, medical education, referrals, and difficult diagnoses. Both non-users and users were surveyed, and non-users were more concerned about potential to disrupt workflow.
**McComb & Bond [****35****]**	Diagnosis	Increasing diagnostic accuracy among junior physicians	Developed and piloted a web-based crowdsourcing software to enable junior doctors to upload cases and receive feedback from expert physicians, with an element of gamification using reward points.	The web interface improved diagnostic ability of junior clinicians, but senior clinicians were less actively involved, due to workload, time, availability and reluctance to embrace the new technology.
**Freifeld et al. [****36****]**	Surveillance	Review of participatory epidemiology	The author provides a review of participatory epidemiology, including FrontlineSMS, Ushahidi, GeoChat, Asthmapolis, and Outbreaks Near Me.	While, at the time of this review, participatory epidemiology platforms were relatively new, there were already palatable benefits.
**Chunara et al. [****37****]**	Surveillance	Online self-reported influenza	Volunteer users filled in a short survey regarding flu symptoms, and enrolling family members. Volunteers enter information weekly, and a map of influenza is available to them.	9300 users in August 2012 throughout the US.
**Michael & Geleta [****38****]**	Surveillance	Global disease surveillance	Describes a smartphone app, Click Clinica, to increase the identification of infectious diseases globally. App contains clinical guidelines, and questions to confirm diagnosis and resistance information.	When ‘live’ for one month, app had already been downloaded over 1000 times and 600 disease notifications had been added. Data was treated as most trusted depending on information provided by submitter (ie, if email, contact details submitted).
**Qureshi et al. [****39****]**	Surveillance	Disease outbreak monitoring	Uses Lady Health Workers in rural areas of Pakistan to report via SMS health information to an electronic disease monitoring system (Jaroka TeleHealthcare System), which provides geospatial location of patients for doctors, medical experts and health officials.	The program was able to display regional patterns for diseases, as well as a disease outbreak that was due to a mass migration of internally displaced persons. The authors reported that the program helps identify whether an epidemic is imminent.
**Lwin et al. [****40****]**	Surveillance	Dengue surveillance	Reports on an app, “Mo-Buzz,” which contains predictive surveillance, civic engagement, and health communication. Citizens use the app or social media to report breeding sites, symptoms and mosquito bites. Using this information, tailored health messages are delivered to individuals living in hot spots. Predictive surveillance predicts outbreaks using this information, combined with weather and other data.	The paper discusses some difficulties with the app, including verifying images due to clarity and receiving multiple images/submissions of the same breeding site. It does not report on the impact of the app on dengue outbreaks.
**Chunara et al. [****41****]**	Surveillance	Malaria surveillance in India	Amazon Mechanical Turk was used to solicit self-reports about malaria diagnosis and related information.	Authors gained information of distribution of malaria species in India, and estimated burden, which coincided with official public health reports.
**Freeman [****42****]**	Identifying erroneous global burden of disease estimates	Surveillance	A crowdsourcing platform was designed, comparing the effect of gamification, to identify erroneous estimates in the global burden of disease database.	Overall, the classifications were matched to a GBD expert 86% of the time. Adding gamification increased accuracy significantly, with gamified users identifying 1.7 times more trends than those using a standard (non-gamified) interface.
**Harrison et al. [****43****]**	Nutrition	Restaurant reviews to identify foodborne illness	Used poor Yelp reviews, specifically those using the words sick, vomit, diarrhea, or food poisoning, to identify food poisoning in New York City restaurants	3 cases of outbreaks met the Department of Health and Mental Hygiene criteria for a food outbreak that were previously unreported.
**Kang et al. [****44****]**	Nutrition	Using Yelp reviews to correlated with failed hygiene inspections	Authors review Yelp, examining whether reviews are correlated with failing hygiene inspections.	The authors find that poor Yelp reviews are correlated with having failed hygiene inspections in Seattle.
**Dunford et al. [****45****]**	Nutrition	Healthier food choices using an app	An app, FoodSwitch, uses crowdsourced submissions of food products in Australia. Crowdsourced submissions are scanned by SKU and then labelled red, green, or yellow to make it easier for consumers to identify healthy foods.	FoodSwitch has been downloaded by 400 000 users and more than 30 000 crowdsourced products have been added to the app.
**Noronha et al. [****46****]**	Nutrition	Nutritional analyses using photos of food – “PlateMate”	Uses Amazon Mechanical Turk, and a step-by-step process to estimate calories, fat, carbohydrates, and protein. First, every food item in a photo is tagged, then identified, then measured, each in a separate HIT.	The application’s error rate was not significantly different from MealSnap (another application) or dieticians. Challenges identified include tagging the entire food item (otherwise it may only be partially measured at a latter stage), or correctly identifying the food item.
**Turner-McGrievy et al. [****47****]**	Nutrition	Nutritional analysis of photos of food – “Eatery App”	Users in the Eatery App post a photo of food, asking other users how healthy it is, and receive crowdsourcing ratings. The goal is to modify diets based on the feedback.	Overall, peer and expert ratings were highly correlated across the US and Europe. Several food categories led to higher healthiness scores among peers (fruit, vegetables, whole grains, legumes, nuts and seeds) and lower healthiness scores among peers (fast food, refined grains, red meat, cheese, savory snacks, desserts, and sugar-sweetened beverages)
**Moorhead et al. [****48****]**	Nutrition	Identifying calories in meals using a smartphone	A mobile application was used to pilot the feasibility of a smartphone app for crowdsourcing with non-experts to identify calories. Training was provided to non-experts, who were asked a month later to estimate calories of food using a photo. A crowd of experts and non-experts was investigated.	Both the crowd of experts and the crowd of non-experts outperformed individual experts or individual non-experts. The expert group estimated the calories significantly more accurately than the non-expert group.
**Bevelander et al. [****49****]**	Nutrition	Predictors of obesity	Participants recruited via reddit, and asked to pose and answer questions regarding childhood predictors of adult BMI for the purpose of generating predictors for a statistical model. Users answered previous questions and posed new ones.	Final sample of 532, 56 new questions identified, 16 of which were highly correlated with adult obesity. Exploratory factor analysis identified 4 factors (home environment, psychosocial well-being, healthy lifestyle, and family history and biological factors). Study identified well-known predictors, but also predictors that had not been well-studied previously. Data collection was rapid.
**Bongard et al. [****50****]**	Nutrition	Predictors of behavioural outcomes	Participants recruited via reddit were asked to pose and answer questions regarding predictors of adult obesity and energy consumptions. The questions and their answers were used to develop predictors in a statistical model.	Despite having a low number of participants in the energy sample, authors were able to develop a predictive model that showed that number of adults in the home and ownership of hot water in the home and an electric heater were predictive. The predictive model for BMI showed demographic, social, economic, genetic, psychological, dietary, and physical-activity related factors.
**Patel et al. [****51****]**	Public Health & Environment	Measuring second-hang smoking in vehicles	Developed an app to be used while driving (by passengers) to measure smoking in other vehicles. Smartphone collects data on number of cars passing (denominator) and user inputs when he/she sees a person smoking in the car, and if so, whether there are other occupants and if occupants are children	A smoking prevalence of was 2.9% in New Zealand, was collected from 66 registered users. These results were similar to a study in 2011.
**Ilakkuvan et al.[****52****]**	Public Health & Environment	Point-of-Sale Tobacco	Uses Amazon Mechanical Turk and image annotation, with micro-tasks (using a zoom feature) to identify point-of-sale tobacco advertising, and compared to field-raters.	Found excellent inter-rater agreement, with AUC averaging over 0.95 (with sensitivity analyses). Author recommends further testing of photograph annotation tools in future work.
**Kim et al. [****53****]**	Public Health & Environment	Point-of-Sale Tobacco	Authors used Gigwalk, which is a mobile crowdsourcing application, to request workers to physically conduct point-of-sale tobacco monitoring. Workers were provided with a manual, but no training, and their work was compared to trained data collectors.	There was extremely high agreement between the crowd and trained data collectors on most measures, so much so that kappa couldn’t be computed in some instances as agreement was perfect.
**Hipp [****54****]**	Public Heath & Environment	Built environment surveillance	Used crowdsourcing to annotate and evaluate captured scenes from 23 000 webcams.	Once annotated, study found changes in behaviour after changes in built environment.
**Castell et al. [****55****]**	Public Health & Environment	Air pollution sensors	Authors describe two projects where sensors are provided to citizens and linked to smartphones, measuring air pollution. The hope is that the sensors will change behaviour patterns, causing citizens to avoid polluted areas, while also producing a map of pollution for cities in which sensors are in use.	The results of these projects were not described.
**Turner et al. [****56****]**	Public Health & Environment	Testing multilingual health promotion	Using Amazon Mechanical Turk, the authors tested promotional health materials with both native English and native Spanish speakers.	Authors were able to reach a more diverse population than with traditional data collection methods, more quickly, and for less cost. They were able to gain more nuanced suggestions to tailor their materials to different populations.
**Hildebrand et al. [****57****]**	Public Health & Environment	Including youth perspectives in HIV/AIDS messaging	A website, CrowdOutAIDS.org, was created to enable youth to be involved in shaping policies, to set priorities and influence actions at UNAIDS. The website intended to connect a community of young people and collect their experiences and ideas and to provide a means to synthesize the information collected from youth globally. Questions were asked in community and online forums in order to ensure youth without internet access were able to participate.	UNAIDS was able to collect information across the globe, highlight similarities and differences from youth both globally and within regions, and to enable youth to influence their policy.
**Merchant et al. [****19****,****20****]**	Public Health & Environment	Mapping Automated External Defibrillators (AEDs)	Developed a crowdsourcing challenge to map AEDs in Philadelphia, which was advertised on TV and radio. Contestants registered via Web or an app, and photographed AED locations (along with AED information) around the city for a chance to win US$ 10 000 prize.	Study lasted 8 weeks. 313 teams and individuals participated and those >40 submitted more entries than younger participants. 1429 submissions were received.
**Tucker et al. [****58****]**	Public Health & Environment	Contest for promotional videos HIV testing programs	Authors launched a contest for promotional videos to encourage HIV testing in China.	Seven eligible videos were received in 2 mo. Videos were judged on reaching untested individuals, engaging the community, and creating excitement around HIV testing.
**Bow et al. [****59****]**	Education	Study materials for medical students	The authors used Google Drive and Java to enable students in the preclinical medicine program to continuously submit and collaboratively edit study questions throughout the course. Prior to the exam, Java turned the study questions into flashcards.	16 150 study questions were created, and the students in that year outperformed students of the previous year in all exams.
**Plenge et al. [****60****]**	Genetics	Genetic prediction of Rheumatoid Arthritis	Authors describe a challenge by DREAM and SAGE, which is a crowdsourcing competition, to develop genetic predictors of response to immunosuppressive therapy in rheumatoid arthritis.	N/A (description was the challenge, but not the results)
**Ewing et al. [****61****]**	Genetics	Detecting somatic mutations from cancer genomes	Describes a DREAM challenge, which is a crowdsourcing competition, to detect somatic mutations from cancer genomes. Employed Google Cloud, and had a public, real-time leaderboard.	Received 248 submissions from 21 teams over approximately 6 mo. The leaderboard enabled teams to improve submissions once they had an initial performance estimate. Finally, authors aggregated submissions of best-performing teams.
**Loguercio et al. [****62****]**	Genetics	Human gene annotation	Dizeez is a crowdsourcing game where players match genes with a clue of the disease; players receive points for selecting the correct disease-gene match. Players can select a specific disease area or protein family. Annotations that are reported across multiple players receive the highest confidence scores.	In 9 mo, 6, 941 unique gene-disease assertions were generated from Dizeez; 2137 were not found in any gene-disease databases (OMIM, PharmGKB, or Gene Wiki). 17 of these associations occurred more than 7 times; these were statistically significant. Authors examined these through a manual literature search and found evidence 14.
**Burger et al. [****63****]**	Genetics	Gene mutation relations	Authors used Amazon Mechanical Turk to judge associations between genes and mutations. Genes were taken from the GenNorm system, and mutations from the Extractor of Mutations system. Genes and mutations mentioned in Pubmed were included, and Turkers were provided with the abstract(s) mentioning the gene and mutations and had to judge if they were related.	The authors explored quality control methods, including repeating experiments on the same HIT and aggregating those results, and eliminating any Turker whose performance fell below 50% accuracy on control items. When these were implemented, accuracy of 89.9% was achieved for a cost of US$ 0.76
**Kido & Swan [****64****]**	Genetics	Exploring relationship between genes and social intelligence	Using results from personal genomics (My Quantified Self), and through tracking personal behavior, the authors explore the relation between the OXTR gene and social intelligence using personality testing	The authors’ results were not statistically significant and need more power; however, the authors’ initial results were in a different direction than hypothesized. Individuals with the AG genotype have lower EQ/IRI values than those with AA, and that an increase in the A allele’s frequency corresponds to decreased optimism.
**Krantz & Berg [****65****]**	Genetics	Incidental findings in GWAS studies	Authors propose using crowdsourcing to solve the problem of reporting incidental findings to populations who have participated in GWAS studies, given that new knowledge of genetic diseases is being discovered.	Proposed system: authors propose a binning system, where crowd sorts findings into clinically actionable, clinically valid but not actionable, or no known clinical significance.
**Shapiro et al. [****66****]**	Psychology	Investigating whether Amazon Mechanical Turk is applicable for mental health	Amazon Mechanical Turk was used, restricted to US residents, to explore whether an AMT population would be a viable research tool for mental health studies. Participants were followed up one week later. Fabrication of mental health symptoms was investigated.	The authors found that Turkers were younger, more educated, white, and more likely to be middle-class compared to the general population. The frequency of trauma exposure and depression. A high proportion of Turkers had clinically relevant anxiety symptoms, but this mirrors previous studies of active internet users. The data were deemed reliable, but authors recommend similar studies in other countries.
**De Choudhury et al. [****67****]**	Psychology	Measuring depression in populations via social media	Uses Amazon Mechanical Turk to obtain a survey on depression, and a self-report of history of depression. The Turkers could opt-in to share their social media handles. Handles that were shared were data mined within a three-month period.	The authors characterize differences between depressed and non-depressed individuals, including time of posting, emotion, linguistic style, engagement and ego-network. These are used to create a social media depression index that could be used to predict risk of depression based on social media posts for other users.
**Hong et al. [****68****]**	Psychology	Advice for people living with autism	Authors wanted to explore using crowdsourcing for advice with people with autism, compared to in-group advice for the same. Questions were selected from online help forums, and authors uploaded those questions to Amazon Mechanical Turk.	Authors received responses within hours, and paid US$ 90 for 400 responses. Out-group responders (those without autism) were more direct in advice, provided superior informational value, and more helpful answers than the in-group responses.
**Yang & Srinivasan [****69****]**	Psychology	Social media surveillance	Used Amazon Mechanical Turk workers to write 20 alternative sentences for each life satisfaction statement. Statements were then used to data mine Twitter.	1000 statements were collected in 5 d for less than US $10.
**Love et al. [****70****]**	General medicine/ Other	Collateral damage of breast cancer treatment	A webpage was designed to collect information regarding collateral damage of breast cancer treatment from survivors.	1191 responses were collected. While many issues reported were known side effects, some issues were not commonly reported.
**Carter et al. [****71****]**	General medicine/ Other	Ovarian cancer awareness	Using Amazon Mechanical Turk, the authors conduct a survey to explore awareness of ovarian cancer, using breast cancer as a control.	Knowledge of ovarian cancer was low among the population studied (which was a US population).
**Good et al. [****72****]**	General medicine/ Other	Gene selection for breast cancer survival	Created a game called “the Cure” which trained players prior to entering the main gaming area. Once in this area, gamers play against an automated opponent, selecting genes in a decision tree classifier, with the aim of surviving.	The authors divided players into ‘experts’ and ‘inexperienced’ and found that the expert group and considering both groups together significantly enriched knowledge for cancer related diseases, while the inexperienced group’s results did not.
**Yu et al. [****73****]**	General medicine/ Other	Evaluation of medical pictograms	Amazon Mechanical Turk was used to obtain judgement of meaning behind medical pictograms.	Comprehensibility scores were calculated, which ranged from 45% to 98%, and correlated strongly to those in another study uses oral responses with the same pictograms. Misinterpretations were judged to be based on errors within the pictograms themselves, not with the Turkers’ abilities.
**Seifert et al. [****74****]**	General medicine/ Other	Fact extraction from scientific literature	Authors present a conceptual framework for scientific fact extraction from literature in different disciplines, to assist researchers who are conducting cross-disciplinary research.	N/A
**Dumitrache [****75****]**	General medicine/ Other	Extracting annotation from medical text	“Dr. Detective” is a game that uses medical experts as a crowd, and is designed to extract annotation and solve disagreements in medical text.	The results from the crowd were comparable to those of natural language processing parser.
**Parry & Tsai [****76****]** **Mortensen et al. [****77****]**	General medicine/ Other	Semantic tagging of medical documents	Used CrowdFlower, and uploaded SNOMED CT relationships and a definition; the crowd was asked whether this was true or false. Experts were also asked to evaluate relationships.	200 SNOMED CT relationships were evaluated (each by 25 workers). The experts and crowd responses were nearly indistinguishable. Errors were identified, which is concerning regarding the biomedical ontologies within SNOMED CT.
**Zhai et al. [****78****]**	General medicine/ Other	Natural language processing	Using CrowdFlower, crowdsourcing medication names, types and linked attributes of clinical trials that were randomly selected from ClinicalTrials.gov	High agreement between crowd’s annotations for medication names and types, correction of previous annotations and linking medications with their attributes. The authors found that simple voting provided the best form of aggregation.
**Gottlieb et al. [****79****]**	General medicine/ Other	Adverse drug reactions	Used Amazon Mechanical Turk to rank severity of adverse drug reactions (ADRs), which were retrieved from the SIDER2 database. Turkers were provided with 10 pairwise comparisons of ADRs and were asked to select which is worse.	ADRs ranked as more serious by Turkers were also associated with more deaths in the FDA adverse events reporting system.
**Khare et al. [****5****]**	General medicine/ Other	Drug indication curation	Used Amazon Mechanical Turk to curate drugs indications. HITs were simplified by asking Turkers to make a judgement of whether a drug label is indicated for a disease, which is highlighted.	3000 HITs were posted from 706 drug labels in 8 h. The aggregated accuracy was 96%, and the total cost was US$ 1.75 per drug label, which is substantially less expensive that traditional alternatives.
**Dasgupta et al. [****80****]**	General medicine/ Other	Black market prices for prescription opioids	Uses StreetRx (a crowdsourcing website) to obtain prices of prescription opioids. Visitors to the website anonymously post the price they paid for prescription opioids and where they were purchased (and are able to see similar purchases and prices).	954 reports were obtained through the website. These were compared to prices provided through law enforcements and through the dark web. The prices were highly correlated between the three.
**Maki & Cohstaedt [****81****]**	General medicine/ Other	Physical crowdsourcing of mosquito samples	Authors collected physical samples of diptera culcidae mosquito through crowdsourcing methods.	Authors received 110 shipped samples of mosquitos, 60% of which came from individuals unknown to laboratory members. Mosquitos came from areas that were difficult to reach.
**McInerney et al. [****82****]**	General medicine/ Other	Logistical deliveries via crowdsourcing	Authors propose a distribution method using the mobility of the local population, and using information gained by cell towers. Participants would exchange packages at a point they normally visit, at a time they normally visit it.	Authors piloted their method, but did not describe it well.
**Maier-Hein et al. [****83****]**	General medicine/ Other	Reference correspondence in endoscopic images during minimally invasive surgery	Used Amazon Mechanical Turk to find sets of corresponding points in endoscopic images, the results of which were compared to medical students and experts.	The experiment took 77 ± 16 min for 100 HITs. The authors note that 10 000 annotations could be generated in 24 h. Using a clustered analysis, the authors obtained an accuracy that outperformed 4 of 5 experts.
**Behrend et al. [****84****]**	General medicine/ Other	Viability of crowdsourcing for organizational survey research	Used Amazon Mechanical Turk to collect basic demographic information and information on internet knowledge, computer attitudes and knowledge, goal orientation, and personality. The results were compared to a control, which was a traditional psychology participant pool.	Both samples were similar in demographic characteristics; however, the crowdsourcing sample was more diverse in education, employment status and profession. There was slightly better social desirability and reliability in the crowdsourced data. The authors conclude that crowdsourcing is a good data pool for organizational research.
**Carlson [****85****]**	General medicine/ Other	Clinical trial protocols	A clinical trial protocol was crowdsourced for input from physicians and patients.	43 physicians and 33 patients took part in the crowdsourcing process to inform development of the clinical trial’s protocol.
**Villaroel [****86****]**	General medicine/ Other	Health care priority setting	Uses Amazon Mechanical Turk to identify health care priorities, asking ‘what should be your priority when treating disease.’ Turkers were asked to distribute 100 points among 5/8 questions (which were randomly assigned)	Dimensions identified include: scale of disease, household financial effects, social equity, cost-effectiveness, spillover effects. It is unclear from the manuscript which rated most important.
**Meisel et al. [****87****]**	General medicine/ Other	Healthcare costs	Suggests crowdsourcing health care costs as a response to higher health care costs for out-of-pocket health care consumers than those insured in the US. Specifically, suggests hosting a website where users can gain access by posting their (de-identified) medical bills.	N/A

### Diagnosis

Diagnosis was the most common usage of crowdsourcing in health. Crowdsourcing has been used multiple times for diagnosing malaria, specifically, and then for grading images in order to diagnose various conditions and diseases [24-28,30-32,88]. It has also been used to assist physicians in diagnosing conditions [34,35].

Three articles described the BioGames project, where laypersons were able to diagnose red blood cells (RBCs) infected with malaria [24-26]. Mavandadi et al. argue that rapid diagnostics for malaria are expensive, unreliable due to heat/stability issues and not trusted by health care workers (HCWs) in low- and middle-income countries and that gamification of malaria diagnosis could improve the management of malarial fevers, be a better use of funding and reduce drug stock-outs [24]. BioGames was available by Android and online. Gamers were given a tutorial, then in the game had a syringe to ‘kill’ infected cells and ‘collect’ healthy ones. The gamers reached an accuracy of 99% (95.1% sensitivity, 99.4% specificity), with the highest level of accuracy being with the largest crowd; however, the authors believe that with a larger crowd, a hybrid algorithm of machine learning and crowdsourcing would perform optimally. Ozcan argues using this method to create gold standard image libraries, for telepathology, point-of-care diagnostics in other conditions and also, to train HCWs in low-income settings [25]. Using the same platform, Feng et al. report on training an education module of the game. The authors used positively or negatively marked cells and had training and diagnostician versions so users could see progress, review misdiagnosed cells and view their accuracy in comparison to their peers. The authors found it was easiest to diagnose negative cells and proposed that in future, laypersons or machines could pre-screen negative cells and send questionable and positive cells to experts for diagnosis. The BioGames project had >2150 gamers participate from over 77 countries, providing over 1.5 million diagnoses [26]. A second malaria diagnostic project, MalariaSpot, utilised gamers over a website to also diagnose malaria in RBCs online. In the span of a month, this project had participants in over 95 countries, and over 12 000 games played [27]. Participants were asked to tag as many malaria parasites as possible in 1 minute. Like BioGames, the accuracy of MalariaSpot was also over 99%.

Crowdsourcing has also been used to diagnose conditions through grading images. Mitry et al. used the Amazon Mechanical Turk (AMT) platform, which is an online crowdsourcing platform that pays users micropayments for small tasks, to grade glaucoma images. The crowd performing the tasks on AMT are called “Turkers”. The authors obtained 2540 classifications of 127 disc images, with 83.22% sensitivity, but only 35%-43% specificity and suggested further micro-izing tasks to improve accuracy [28]. AMT was employed in another study to grade images to screen for diabetic retinopathy, with three phases of grading. The authors found that Turkers were able to sufficiently determine normal vs abnormal, but had trouble grading the severity of retinopathy; however, sensitivity for whether retinopathy was present was 100% at all stages [29]. Another study used a website to gather a crowd’s scores of estrogen receptor in breast cancer tumour tissue. A portion of the tissues was previously scored by a pathologist, allowing the authors to assign users a ‘user performance scale’ and a reliability/trust score for the crowd. The authors found the crowd’s ability was similar to that of a trained pathologists [30]. Gehl et al. used a non-internet based crowdsourcing study to explore skin self-examination (SSE) for atypical moles. The authors recruited 500 participants from a mall, administered a pre-test and post-test and provided each participant with basic SSE techniques. The analysis used a threshold of 19% of participants to identify the mole as abnormal for it to be considered as such. Using this threshold, the participants correctly identified 90% of the melanomas and 72% of the non-melanomas [31]. Xiang et al. attempted to use crowdsourcing to answer the need for a scarcity in medical imaging specialists outside major cities in China. The authors recruited 13 students in 2nd and 3rd year of medical school with a medical imaging major. The average accuracy for the individual was 39.54%; however, the authors were able to increase the accuracy using crowdsourcing algorithms to 56%, and found that machine learning algorithms performed higher. The authors noted that since all the participants were from the same department in the same medical school, the lack of diversity and experience may have contributed to the poor results [32].

Three articles described used crowdsourcing to either assist physicians in providing a diagnosis or to replace physicians in providing a diagnosis. Cheng et al. investigated the feasibility of three crowdsourcing platforms to provide diagnoses: volunteer platforms (ie, Yahoo! Answers), Amazon Mechanical Turk (AMT), and O’Desk. They assessed cases of easy, medium and hard levels of difficulty. The case studies of hardest level of difficulty were taken from CrowdMed.com, which is a website which enables patients with “mystery” diseases to provide their detailed medical information to a crowd of medical experts (including specialists and medical students) who will submit their answers, and the correct diagnostician receiving a reward. The easy and medium levels were taken from another paper, which posted medical questions on Facebook to see if Facebook friends were able to answer [33,89]. The authors’ attempts at posting on the volunteer sites failed, as they did not receive responses. On AMT, the Turkers were able to solve many of the easy cases but had trouble with the medium difficulty cases, although the authors stated that these may have been poorly described. None of the CrowdMed cases were answered correctly by AMT workers. O’Desk is a crowdsourcing platform that contracts employees, and the authors hired health care professionals. All the easy level questions were answered correctly by O’Desk, but each only answered one medium level case correctly. The original O’Desk workers declined to answer the CrowdMed cases, expressing uncertainty; the second contracted O’Desk workers were unable to answer correctly.

The DocCHIRP (Crowdsourcing Health Information Retrieval Protocol for Doctors) is a mobile application that helps clinician’s problem solving at the point-of-care. Sims and colleagues presented the experiences of clinicians using the application. 78% of clinicians using the application reported benefit on routine patient care, medical education and accurate referrals, as well as diagnosing unusual cases. Some concerns reported by clinicians included lowered productivity, due to responding to the application, and interference with ‘off the clock’ time, though the latter was only reported among non-users [34]. McComb and Bond also reported on an application that assists clinicians in making diagnoses. Their application, called CoDiagnose, has junior doctors upload case information and receive feedback from a crowd of expert clinicians and it features a built-in e-Learning component. The junior doctors’ diagnostic accuracy improved 14% with the use of the application; however, the authors reported a lack of enthusiasm on part of the expert clinicians about participating [35].

### Surveillance

Another very common purpose to which crowdsourcing is used in health is surveillance, both in the context of research and in emergency situations for programming. A number of articles described pilots or projects that employed crowdsourcing for health surveillance [36-42]. As of 2013, 70% of the world's population carried a mobile phone [90], making surveillance through mHealth a promising avenue.

Freifeld and colleagues reviewed a number of crowdsourcing platforms that have been used for community surveillance and participatory epidemiology. Frontline SMS, also called Frontline SMS Medic, enables users to request needs via SMS. It has been used in Malawi, Burundi, Bangladesh and Honduras. Ushahidi is an open source crowdsourcing application that collects individual reports via web, SMS, and email. It can classify, translate and geotag results. Ushahidi was initially created in response to election violence in Kenya, but it has been used most famously in the aftermath of the Haitian earthquake. It has also been deployed in Uganda, Malawi, Afghanistan, and Zambia. Ushahidi also has a feature for collecting voice reports, which is essential for people who are not literate. Geochat is another crowdsourcing application. It aims to aid in faster and more coordinated responses to disease outbreaks and natural disasters. Team members use the application to communicate their location through SMS, email and web. This information synchronises on all users’ devices. The application has been launched in Thailand and Cambodia [36].

Asthmapolis is a GPS-enabled inhaler that is linked to a user’s phone and tracks asthma attacks. The application compiles the information from those using its inhalers and generates a risk map for environmental triggers [36]. Freifeld et al. also reported on two other crowdsourcing applications, HealthMap and OutbreaksNearMe, which mapped influenza in the United States using submissions by laypersons. Chunara et al. also reports on a similar tool, FluNearYou, which maps influenza outbreaks using submissions by laypersons and generates a map to identify outbreaks [37].

ClickClinica is an application that was developed to provide General Practitioners (GPs) and Medical Doctors (MDs) indexed guidelines for diseases. Currently, GPs and MDs use the application to diagnose diseases by asking questions about the condition of the patient. The submitted data are graded by the quality of the user information, ie, if the submitter has a medical ID or an institutional email. Over 1000 MDs across the world have been using the application, despite it not being marketed. The application can also be used to increase recruitment for clinical studies through GIS notification of nearby, relevant studies. The authors suggested that this application could be developed into real-time global surveillance based on usage [38].

Qureshi and colleagues described the Jaroka Tele-Health System, which employed Lady Health Workers in rural Pakistan to use SMS/MMS to register patients, report symptoms, acquire prescriptions and connect to specialists. The resulting data was then used to track disease spread and the authors have been able to use the visualised data via crude numbers or rates and identify clustering; the authors found higher rates of disease during times of migration and with internally displaced persons, which could be explained by poor sanitation and overpopulation. They also found increased rates of hypertension in women in these populations [39].

Lwin et al. designed a participatory epidemiology application, called Mo Buzz, in Sri Lanka to combat dengue. Mo Buzz has three components: (i) predictive surveillance; (ii) civic engagement; and, (iii) health communication. The predictive surveillance component uses a machine learning algorithm to determine weather, vector and human data in the form of hotspot maps for the public and for health officials. The civic engagement component involves people reporting breeding sites, symptoms, and bites; these are reflected on the hotspot map. Finally, this information is communicated to the public and to health officials [40].

Chunara et al. use AMT to survey the malaria prevalence in India. Turkers are asked questions regarding malaria symptoms, date of onset, the malaria status of their household members and their awareness of malaria in their communities. The study found that diagnosis peaked in August and this correlated with official reports [41].

A recent study using Global Burden of Disease (GBD) data examined creating a game to scan the GBD database for errors, as algorithms currently used are imperfect. While participants were staff from the Institute for Health Metrics and Evaluation at the University of Washington, and thus not laypersons with no background knowledge, the author found that using gamification increased the accuracy of results by 1.7 times [42]. As only 4% of infectious diseases have been comprehensively mapped, innovative solutions such as AMT and other crowdsourcing applications detailed above may be useful in providing infectious disease mapping and surveillance [91].

### Nutrition

In the area of nutrition, articles employing crowdsourcing focused on food safety, food labelling, assessing how healthy the meals were, and identifying predictors of obesity [43-50]. Two articles used Yelp reviews to assess food safety in restaurants, one in New York City and the other in Seattle [43,44]. In New York City, the Department of Health and Mental Hygiene received data from Yelp and used computer algorithms to scan the data for probable food poisoning cases. These cases were then reviewed by a foodborne epidemiologist, and probable cases were requested for interview. Three outbreaks were discovered by the study [43]. The second article, which uses Yelp reviews for Seattle, tested whether Yelp reviews would be able to predict whether a restaurant would fail its health inspection. The authors found that the Yelp reviews were 82% accurate predictors for restaurants that would fail health inspections [44].

As food packaging is often complicated and difficult to understand for consumers, Dunford and colleagues created a traffic light application that is populated through crowdsourced submissions in order to enable people in Australia to make healthier choices about their diets [45]. Their application is called “FoodSwitch Australia.” Three other articles published efforts to ensure consumers were able to make healthy decisions about the food they eat. Noronha and colleagues, Turner-McGrievy and colleagues and Moorhead and colleagues developed applications to enable crowds to rate the ‘healthiness’ of food being eaten. Noronha et al.’s application, “Platemate” had participants take a photo before and after their meals and asked the crowd to estimate the calories and nutritional composition. They found the estimates of the crowd did not differ significantly from that of experts [46]. Turner-McGrievy et al.’s “EateryApp” had the crowd rating the healthiness of photos of food after 1.5 hours of training. The authors also compared their ratings to those of experts and found a strong correlation between the ratings (r = 0.88, *P* < 0.001) [47]. Moorhead also used photos to have a crowd estimate the calories in food, but also developed personalised messages for prevention and management of obesity. They piloted their application with a crowd of 12 non-experts and 12 experts. In both cases, the group estimates were more accurate than any individual estimate [48].

Finally, two articles reported on using crowdsourcing to develop predictors of obesity in a statistical model [49,50]. Both articles used reddit to recruit participants and had participants initially answering questions commonly known to be predictors of obesity. Then, they posed new questions, which the group as a whole would answer. Bevelander et al. found that some participants identified examples that were not well documented in literature and suggested that those could be possible new directions for future research. The study also found that only about 7% of participants posed new questions; the remaining participants answered questions only.

### Public health and environment

Crowdsourcing has been used in public health for research in the areas of tobacco control, physical activity and built environment, environmental health, to shape messaging and for public health related contests [51-58].

Patel et al. created an application to measure the prevalence of smoking in vehicles. The application had a mechanical counter to track passing vehicles, thus providing the denominator, and the person using the application would record each instance of a person smoking in the vehicle. The authors found a prevalence of 2.9% and had 66 users [51]. Two articles used crowdsourcing for point-of-sale tobacco (POS) monitoring. Ilakkuvan and colleagues used AMT for image annotation. The authors were testing image annotation, rather than monitoring per se, and found that image annotation improved when Turkers were provided with microtasks and given the option to zoom in on photos [52]. The final article examining POS tobacco use physically deployed their crowd to the locations under surveillance. The crowd photographed the stores and answered questions. The authors found high agreement on what the stores sold, but poor agreement on promotions; however, authors noted this could be due to the crowdsourced visitors visiting the stores at different times than the trained experts, when the stores simply had different promotions [53].

Hipp and colleagues used webcams in the United States to capture changes after altering the built environment, for example, by adding a crosswalk or adding a bike lane. The authors then used AMT to annotate the webcam images for the pedestrian and cyclist traffic before and after the changes to the built environment. This was done in order to determine the impact the alteration has had on people’s behaviours. The authors found that AMT was a successful method for image annotation and that there were measurable changes after the built environment was altered [54]. Castell et al. used physical crowdsourcing to explore environmental health, and they hoped to have an impact on the health, too. The authors created CITI-SENSE and CITI-SENSE-MOB, two applications which used sensors that were attached to mobile phones that obtained individual-level data on air quality pollution that were GPS-tagged. These data provided users with a map of where pollution was the worst, so they could avoid it [55].

Turner and colleagues employed AMT to test multilingual promotional dental materials (in English and Spanish). They were able to receive feedback from almost 400 Turkers in less than 2 weeks and received especially valuable feedback from the Spanish-speaking Turkers regarding the cultural appropriateness and dialects of their messaging [56]. An additional public health messaging project that used crowdsourcing was CrowdoutAIDS, which was a large campaign by the UNAIDS Secretariat that had included youth, both online and offline, from 79 countries in shaping UNAIDS messaging and their priorities for sexual health [57].

Another way crowdsourcing has been used in public health is through contests to draw attention to important causes and promote public engagement. In Philadelphia, a large crowdsourced competition was deployed to bring attention to heart disease through mapping automated external defibrillators (AEDs) [19,20]. Through this contest, 1429 AEDs were located through 313 submissions. Data were validated through GPS, door-to-door and photo verifications. The authors were pleasantly surprised that, despite being a social-media based exercise, many older participants contributed. Another exercise that used a crowdsourcing contest to draw attention to a public health concern asked participants to design and develop videos to promote HIV testing in China. Seven eligible videos were submitted in an eight-week time period [58].

### Education

Bow and colleagues reported using crowdsourcing with their pre-clinical medicine students at Johns Hopkins University to create flashcards to improve studying. The professors had questions and respective answers after lectures available on GoogleDrive, which was shared with the class. Students were able to add to questions, add new questions and add to answers. The questions were changed to flashcards using Java, to assist students as study aids. The students’ grades improved in comparison with students from the previous year [59]. Another study stated that crowdsourcing could help educators grade students’ assignments more fairly, but did not elaborate [92].

### Genetics

In genetics, crowdsourcing has been used for challenges in genetic research, for matching genes to mutations, to identify novel hypotheses through crowdsourced data, and it was proposed as a solution for incidental findings in genomics studies [60-65].

Sage and Dream are two organisations that heavily use crowdsourcing in genetics research. Plenge et al. report on one of their challenges, which was to develop genetic predictors of a response to immunosuppressive therapy in rheumatoid arthritis, using genome-wide association study (GWAS) data. The challenge was team-based, collaborative and open to both public and private contributors. The team that produced the best predictive model would win the challenge [60]. Ewing and colleagues reported on another Dream challenge, which aimed to identify somatic mutations in cancer genomes. The data for this challenge was distributed via GoogleCloud, and the challenge employed a Leaderboard for a competitive aspect. There were 248 submissions by 21 teams in 157 days [61].

Dizeez is a human gene annotation game where players guess which gene causes which disease, out of four options. The game aims to identify gene-disease associations that are known but not present in structured annotation databases. Authors took the genes selected to be associated with a particular disease by many players for further investigation. The authors note that, unfortunately, when players suggest potentially novel associations, they are ‘punished’ by the game. However, the game was able to successfully identify gene-disease associations [62]. Another gene matching game, EntrezGene, used AMT to match genes from papers and abstracts to their EntrezGene identifier. Turkers were asked to judge whether the gene is associated with a mutation and 20% of the tasks were controls. In the authors’ report, there were problems with the study giving false information to the Turkers but after adjusting for this, the Turkers achieved 82.3% precision [63].

Kido and Swan reported on using crowdsourced data from MyQuantifiedSelf, which is a personal genomics company, in order to test their novel hypothesis, that some genetic profiles would exhibit a natural capacity for social intelligence. The authors combined citizen science as a form of crowdsourcing and the daily tracking of their “MyFinder” application in order to explore the role of genomics (OXTR gene mutations) on personalities. The authors found that their hypothesis did not appear to be valid, that the increase in the frequency of the G allele did not lead to increased optimism; however, an increase in the frequency of the A allele seemed to lead to decreased optimism. The authors stated that further analyses with larger sample sizes needed to be conducted in order to confirm their hypotheses [64].

Finally, as GWAS studies are becoming more prevalent and the probability of incidental findings becomes more likely, Krantz and Berg suggest crowdsourcing as a solution to managing incidental findings. The authors proposed a ‘binning system’ in genetic studies that employs crowdsourcing, such as through AMT, to separate incidental findings into bins based on their current risk to the individual. For example, clinically actionable results would be placed in “bin 1”, while results with a high clinical validity with no actionability would be in “bin 2” and those with no clinical significance would be in “bin 3.” The results in bin 2 would need to be re-scored as new advances in medicine are made [65].

### Psychology

There were four articles published in psychology [66-69]. The first explored the viability of using crowdsourcing, specifically via AMT, for studying mental health issues. The authors assessed misrepresentation, inconsistencies in basic demographic information and clinical symptom reporting. The authors found that AMT workers' mental health mirrors that of the general population, other than social anxiety and satisfaction of life scales. AMT workers have an increased social anxiety, which mirrors other internet-based studies, and lower satisfaction with life scales [66]. A second study used AMT to diagnose major depressive disorder (MDD). It asked users for the Twitter account and data-mined their accounts for one-year prior in order to measure user engagement, egocentric social graph, linguistic style, depressive language use, and their mention of using antidepressants. The control was a standard user class. The authors found lower social activity, greater negative emotion, higher self-attentional focus, increased relational and medicinal concerns and heightened expression of religious thoughts among the MDD group [67].

The third study explored the potential for crowdsourcing to adequately respond to discussions in an autism support group by outsourcing the help questions to AMT and having the responses rated against the in-group answers. The AMT answers were rated as more helpful and AMT was seen as a quick way to provide direct and informal emotional support and to broaden perspectives of the autistic community [68]. The final study combined crowdsourcing and data mining, using AMT to generate alternative Life Quality Statements to enable comprehensive data mining for these statements from Twitter [69].

### General medicine/other

Remaining examples of crowdsourcing were in areas of oncology, medical text, various aspects of drugs, including curation, severity of reactions and even black market prices, examples of physical crowdsourcing and other miscellaneous examples [70-76,78-87,93].

Love and colleagues used crowdsourcing to explore women’s questions regarding collateral damage from breast cancer treatment. Advocacy organisations collected responses to the questions posed. Many of the women who submitted questions complained of fatigue, memory loss, numbness, anxiety or depression [70]. Another study used crowdsourcing to survey the knowledge of the population about ovarian cancer, using breast cancer as a control. The authors used AMT as a platform and found that 56% of those surveyed reported no knowledge of ovarian cancer [71]. A third study employed a crowdsourcing game, ‘the Cure,’ to predict breast cancer survival in order to improve prognostic indicators of breast cancer. Approximately 60% of players were not knowledgeable about breast cancer. The authors reported that both the expert and the all (expert and non-expert combined) set ‘significantly enriched’ knowledge, but that the non-expert group alone did not. The responses of the expert group performed well in a Sage contest as well [72].

As a solution to challenges with health literacy and poor comprehension and adherence to text-based medical instructions, Yu and colleagues used AMT to test picture-based medical instructions using existing pictograms from the Internet. The results showed that semantic concepts were difficult to represent in pictures (ie, take with additional water or take in the AM). Turkers with higher levels of education performed better. Yu et al. suggested future studies should use tailor-designed pictograms and explore the interplay between education and responses as well as the role of culture and different countries in ability to comprehend the pictograms [73]. Seifert and colleagues describe conceptual framework for using AMT with the aim to extract facts from interdisciplinary scientific literature in order to help researchers keep track of overlapping topics across disciplines [74]. Another article described the use of gamification to enable both experts and laypersons to complete text extraction, term categorisation, relation extraction and relation categorisation in games called “Dr. Detective” and “Crowd Watson.” These games employed crowdsourcing through natural language processing (NLP) and relied on inter-annotator agreement [75]. A third example of NLP using crowdsourcing was Parry et al.’s SNOMED CT (Systemised Nomenclature of Medicine-Clinical Terms) which used semantic tagging, similar to Medical Subject Headings (MeSH) terms, in order to help clinicians code free-text documents. SNOMED CT used crowdsourcing, along with a learning algorithm, to imply membership in a particular ontology and the degree of ‘fuzziness’ the member believes the ontology to be; users then rate how related it is [76]. Crowdsourcing was also used to investigate errors in SNOWMED CT, and when compared to experts, their results were comparable [77].

Crowdsourcing has been used in drug research in various ways, including through NLP, ranking the severity of reactions, curating drug indications and to identify black market prices for drugs. Zhai et al. used Crowdflower, which is a crowdsourcing platform similar to AMT, in order to have a crowd perform NLP tasks that identify medication types, names and link these to their attributes. The authors found that there was high agreement between the crowd’s NLP and expert-generated NLP (0.87 for medication names, 0.73 for medication types and 0.90 for linking medication to attributes). There were no significant differences between crowdsourced NLPs and experts after developing a “trust” threshold in the analysis, where the Turker meets a threshold to become “trusted” [78]. Gottlieb and colleagues used AMT to rank the severity of almost 3000 adverse drug reactions (ADRs). The authors found that the ADRs ranked most severe were more correlated with death (r = 0.53) [79]. Khare et al. aimed to use AMT to create a database of unique drug indications, by providing microtasks to Turkers, asking them to differentiate between whether a drug is indicated for a particular condition or the condition is listed for another reason (ie, it is a risk factor, side effect, contraindication, etc.). The crowd achieved over 90% accuracy and over 96% accuracy in identifying drug indications through majority voting [93]. Finally, Dasgupta et al. described the use of crowdsourcing to identify black market prices for opioids through StreetRx. With StreetRx, people were able to anonymously post where, when and the prices that either they or someone they know purchased street drugs for. People were able to access the website without posting, thus drug users were able to see if they were buying their drugs at a fair price. Drug users were motivated to post on the website, as it could prevent them from overpaying for their drugs. The researchers compared the prices on StreetRx to prices reported by law enforcement officials and from a ‘dark web’ website, Silk Road. The researchers found no significant differences, other than for morphine [80].

Three articles reported on physical instances of crowdsourcing. Maki and Cohnstaedt reported on using crowdsourcing to collect physical samples of mosquitos from a mostly trained crowd. Through crowdsourcing, the authors were able to collect mosquitos in “geographically vital, hard-to-access locations” and achieved a 91% response rate [81]. McInerney and colleagues reported on a pilot to employ crowdsourcing and Bayesian modelling to deliver items in low- and middle-income countries. The authors described using cell towers during texts and calls to predict temporality, assessing the number of people to achieve geographic coverage and the feasibility to deliver to rural locations and briefly mention a pilot. Despite an in-depth description of the theory behind their crowdsourcing model and the plans for their pilot, the authors hardly discussed their pilot, except to say that it did not perform well and there were delays in all areas (urban and rural) [82]. Finally, Maier-Hein et al. suggested using crowdsourcing for minimally-invasive surgery that required establishing correspondences and was typically done by a medical expert. The authors found that crowdsourcing was comparable to medical experts and could be used to train algorithms [83].

Similar to the psychology studies which tested whether crowdsourcing could be used to sample a population for psychological studies, Behrend et al. explored whether crowdsourcing would be an appropriate population for organisational research, comparing the demographics of a crowd from AMT to a university population. The authors found that the crowdsourcing population was more diverse professionally, and constituted an attractive pool for organisational research [84].

Carlson described a clinical trial protocol that has been reviewed not only by peers, but by patients, using crowdsourcing. He asserted that the clinical trial protocol was faster, and that using the intellectual capacity of patients in addition to researchers would enable the trial to attract participants [85]. Another author advocated for including the general population in health research, but in shaping their research priorities. Villaroel gave an example of a research priority setting exercise that was done in India using AMT. In this exercise, there were significant differences in priorities of those who did and did not vote in the last election, leading the author to assert that elected officials may not have the entire populations’ interests at heart [86]. Finally, Meisel et al. suggested an innovative solution using crowdsourcing to combat overcharging at US hospitals. As US hospitals often charge different rates for insured and non-insured customers and can charge exorbitant rates for simple procedures, such as US$ 55 000 for an appendectomy, the authors suggested creating a database where consumers post their hospital bills. Membership in this database could be free once the user has posted one bill. This would help maintain accountability and transparency among the hospitals [87].

## DISCUSSION

Many studies focused on using crowdsourcing for diagnostics or for surveillance. Indeed, it appears that crowdsourcing can be uniquely positioned to improve diagnostics and surveillance of illness. Strategies to improve accuracy included employment of machine-learning algorithms [24,32,40,76], gamification [24-27,42], and establishing thresholds for trustworthiness or questions to weed out malicious workers [31,63].

Crowdsourcing has the potential to improve diagnostics two ways; first, employing a crowd of laypersons and experts through gamification of diagnostics, as shown through BioGames and MalariaSpot [24-27] which have proven to be an effective way arrive at an accurate diagnosis without the need for experts. These games were especially beneficial at identifying RBC that were negative for malarial parasites, which could free expert time, enabling experts to spend time confirming positive blood smears. As Feng et al. and Ozcan suggested, BioGames can be used to train HCWs in LMICs to diagnose RBCs for malaria, which is extremely valuable. While these articles focused on malaria diagnostics, it can easily be imagined that a similar game could be made to diagnose other bloodborne pathogens. If such a game had similar success, online gaming through crowdsourcing could be used to complement traditional laboratory diagnostics and laboratory technicians would spend much less time looking at negative blood smears.

Applications that were developed to use a crowd to grade images had less success than those to identify infected blood cells, though Turkers seemed to be proficient at identifying whether there was presence or absence of retinopathy [29] and when authors employed innovative techniques such as trustworthiness scales or thresholds in their analyses, they achieved better results [30,31]. Other strategies to increase accuracy that have been suggested include providing microtasks; Ilakkuvan et al. reported better results after enabling a zoom feature for image annotation and having micro image annotation tasks [52]. This is synonymous with suggestions of conditions under which to use crowdsourcing that were previously reported [8,9,15].

The second way crowdsourcing can be used in diagnostics in by helping clinicians make diagnoses through applications such as DocCHIRP and CoDiagnose, which had promising results. However, both applications appeared to have some push-back from their ‘crowd’ of experts [34,35], which questions their sustainability.

Surveillance was the area in which crowdsourcing has been the most successful at the largest scale. Freifeld et al. listed many successful crowdsourcing surveillance applications that are being used for health care in emergency and disaster response situations, such as Ushahidi, Frontline SMS and Geochat [36]. These studies also lowered barriers to entry (for example, through providing multiple ways to submit data, including through voice to text, providing translation services, etc.), which may have contributed to their success [36]. Many of the surveillance applications include some component of civic engagement, either overtly like Mo-Buzz, which uses its predictive surveillance for health communication or less obviously, by relying on mass submissions from laypersons in order to function [40].

Crowdsourcing was found to be useful to predict poor sanitary conditions and foodborne illnesses based on Yelp reviews [43,44], to assess whether meals were healthy, irrelevant of whether the crowd was formed of experts or laypeople [46,47] and to identify predictors of obesity in statistical models for childhood and adulthood obesity [49,50]. Applications to help people make healthy choices based on where and what they eat are important. Harrison et al. and Kang et al. have shown that Yelp reviews can be used to direct scarce resources from Public Health Departments to direct food inspectors to the most likely the culprits. Applications that show consumers how healthy their meals are could be important not only in obesity prevention, but also to ensure that consumers are eating a well-balanced diet. Bevelander et al. reported that their crowdsourcing exercise identified predictors of obesity that were not found in the literature and that should be explored in future research [49]. This method to identify predictors for statistical models for both well studied and less commonly researched diseases could be especially beneficial before beginning a study, while deciding which data will be collected.

Some of the research that has taken advantage of crowdsourcing in the public health and environmental health would not have been possible without crowdsourcing. For example, Hipp’s characterisation of changes in the built environment’s effect on physical activity, using web cam footage, would not have been possible without crowdsourcing; it would have been impossible to have the hours and hours of webcam footage annotated. The CITI-SENSE and CITI-SENSE-MOB project, which uses mobile phones to obtain individual level data that are GPS tagged to map air pollution, would also be impossible without crowdsourcing.

Crowdsourcing competitions, for public health or for genetics, have proven to be successful. Merchant et al. and Tucker et al. have used crowdsourcing to draw attention to AEDs in Philadelphia and to HIV testing in China, respectively [19,20,58], whereas Sage and Dream organisations have conducted many challenges for genetic research to identify genetic predictors of immunosuppressive therapy in rheumatoid arthritis or to identify somatic mutations in cancer genomes [60,61]. Kido et al. reported on an innovative combination of citizen science and crowdsourcing, using MyQuantifiedSelf, which takes personal genomics and uses a crowd of people who have MyQuantifiedSelf results to fill in personality tests to test the relationship between genetics and personality [64]. Other authors have included laypersons in gamifying genomics, through EntrezGene, which matches genes to their abstract using AMT [63]. Finally, due to the high number of incidental findings being identified in GWAS studies, Krantz and Berg suggested crowdsourcing as a solution, using a large crowd to go through the findings and putting the findings into ‘bins’ depending on their clinical validity and actionability [65].

Crowdsourcing has been used to predict survival of cancer, using both laypersons who were not knowledgeable about cancer and experts (though the expert group performed better), which demonstrated that having some knowledge is important when the subject matter is advanced [70]. However, laypeople have been able to perform to expert-level accuracy in other tasks, such as annotation [78,93]. Diagnostics is an area where crowdsourcing is especially promising, as shown by the malaria studies. Interestingly, the most promising studies in diagnostics employed gamification, which was shown to improve accuracy in an unrelated study [24-27,42]. Authors have explored whether crowds of AMT workers display similar characteristics to the general population and concluded that they are appropriate for organisational research and for psychological research, as they differed minimally [66,67,84].

It is important for future work using crowdsourcing to consider the appropriateness of the crowd being used, to ensure the crowd has the capability and the adequate knowledge and also, to design the task and the method of analysis effectively. Freeman found that using gamification (ie, having crowdsourcing activities linked to a game with rewards, scoreboards or some sort of competition) improved accuracy and, examples provided that have used gamification have been quite successful [42]. Other modes of analysis that have been successful include introducing thresholds and degrees of trustworthiness in order for an individuals’ answers to be included into the crowd’s or for the crowds’ answer to be used [30,31]. It is important to note that not all the research, nor all the successful research, in crowdsourcing involved the Internet. Some of the crowdsourcing studies were done in person or involved sending in physical samples [31,81]. Previous definitions of crowdsourcing necessitated using the internet [2], but use of the internet is not compulsory and this is important to stress, especially in the context of global health where use of the internet may not be accessible to all.

## CONCLUSION

Crowdsourcing as a field is still nascent, with the term having only been coined a decade ago [1]. Despite this, it has been used across numerous disciplines in medicine, from diagnosis and surveillance to nutrition, psychology, and even to crowdsourcing minimally invasive surgery. The wideness of uses demonstrates that crowdsourcing applications have been innovative and adaptable. However, many of the crowdsourcing applications have not been used past pilot phases, with the exception of surveillance applications that are used in disaster and emergency relief. These exceptions demonstrate that it is possible to use crowdsourcing at scale; further efforts are needed to take promising crowdsourcing applications to scale in order to provide accessible health care to more communities and individuals rapidly at a low cost.
